# Behavior Change Techniques Used in Self-Management Interventions Based on mHealth Apps for Adults With Hypertension: Systematic Review and Meta-Analysis of Randomized Controlled Trials

**DOI:** 10.2196/54978

**Published:** 2024-10-22

**Authors:** You Zhou, Si-Jia Li, Ren-Qian Huang, Hao-Ming Ma, Ao-Qi Wang, Xing-Yi Tang, Run-Yuan Pei, Mei-Hua Piao

**Affiliations:** 1 School of Nursing Chinese Academy of Medical Sciences Peking Union Medical College Beijing China; 2 Department of Nursing The First Affiliated Hospital of Zhejiang Chinese Medical University (Zhejiang Provincial Hospital of Chinese Medicine) Hangzhou China; 3 Changhai Clinical Research Unit Changhai Hospital Naval Medical University Shanghai China

**Keywords:** hypertension, mHealth, app, behavior change technique, systematic review, meta-analysis, mobile phone

## Abstract

**Background:**

Hypertension has become an important global public health challenge. Mobile health (mHealth) intervention is a viable strategy to improve outcomes for patients with hypertension. However, evidence on the effect of mHealth app interventions on self-management in patients with hypertension is yet to be updated, and the active ingredients promoting behavior change in interventions remain unclear.

**Objective:**

We aimed to evaluate the effect of mHealth app self-management interventions on blood pressure (BP) management and investigate the use of behavior change techniques (BCTs) in mHealth app interventions.

**Methods:**

We conducted a literature search in 6 electronic databases from January 2009 to October 2023 for studies reporting the application of mHealth apps in self-management interventions. The Cochrane Risk of Bias (version 2) tool for randomized controlled trials was used to assess the quality of the studies. BCTs were coded according to the Taxonomy of BCTs (version 1). The extracted data were analyzed using RevMan5.4 software (Cochrane Collaboration).

**Results:**

We reviewed 20 studies, of which 16 were included in the meta-analysis. In total, 21 different BCTs (mean 8.7, SD 3.8 BCTs) from 12 BCT categories were reported in mHealth app interventions. The most common BCTs were *self-monitoring of outcomes of behavior*, *feedback on outcomes of behavior*, *instruction on how to perform the behavior*, and *pharmacological support*. The mHealth app interventions resulted in a –5.78 mm Hg (95% CI –7.97 mm Hg to –3.59 mm Hg; *P*<.001) reduction in systolic BP and a –3.28 mm Hg (95% CI –4.39 mm Hg to –2.17 mm Hg; *P*<.001) reduction in diastolic BP. The effect of interventions on BP reduction was associated with risk factors, such as hypertension, that were addressed by the mHealth app intervention (multiple risk factors vs a single risk factor: –6.50 mm Hg, 95% CI –9.00 mm Hg to –3.99 mm Hg vs –1.54 mm Hg, 95% CI –4.15 mm Hg to 1.06 mm Hg; *P*=.007); the presence of a theoretical foundation (with vs without behavior change theory: –10.06 mm Hg, 95% CI –16.42 mm Hg to –3.70 mm Hg vs –4.13 mm Hg, 95% CI –5.50 to –2.75 mm Hg; *P*=.07); intervention duration (3 vs ≥6 months: –8.87 mm Hg, 95% CI –10.90 mm Hg to –6.83 mm Hg vs –5.76 mm Hg, 95% CI –8.74 mm Hg to –2.77 mm Hg; *P*=.09); and the number of BCTs (≥11 vs <11 BCTs: –9.68 mm Hg, 95% CI –13.49 mm Hg to –5.87 mm Hg vs –2.88 mm Hg, 95% CI –3.90 mm Hg to –1.86 mm Hg; *P*<.001).

**Conclusions:**

The self-management interventions based on mHealth apps were effective strategies for lowering BP in patients with hypertension. The effect of interventions was influenced by factors related to the study’s intervention design and BCT.

## Introduction

### Background

Hypertension is a chronic disease characterized by persistent elevation of systemic arterial blood pressure (BP) and is considered the most important risk factor for cardiovascular diseases [1]. Epidemiological evidence suggests that approximately 31.1% of adults worldwide had hypertension in 2010 [2]. With the acceleration of population aging and the increase in lifestyle risk factors such as alcohol consumption, obesity, lack of physical activity, and unhealthy diets, the global prevalence of hypertension has shown a significant upward trend [3]. The Lancet Commission on hypertension has stated that primordial, primary, and secondary prevention of hypertension in the life course should be implemented to address the global burden of increased BP [4]. Therefore, people with risk of hypertension and patients with hypertension need to carry out BP management including lifestyle modifications addressing various lifestyle risk factors and medication management after diagnosis.

Mobile health (mHealth) refers to medical and public health practices supported by mobile devices, such as mobile phones, patient monitoring devices, and other wireless devices [5]. Considering its advantages, such as low cost and ease of use, the development of self-management solutions for patients with hypertension based on mHealth apps has become a frontier and hot spot of research. Several meta-analyses have demonstrated that mHealth interventions are effective for patients with hypertension and can improve clinical outcomes [6-11]. For example, a meta-analysis including SMS text messaging, smartphone apps, and website interventions showed that mHealth interventions, particularly smartphone apps, were associated with clinical reductions in systolic BP (SBP) [10]. Another meta-analysis showed that mHealth apps significantly improved medication adherence of patients with hypertension and increased patients’ perceived confidence, treatment self-efficacy, acceptance of technology, and knowledge about health issues [11]. However, all the aforementioned studies lacked further analysis to identify the study design factors and active ingredients in the interventions or apps that promote self-management effectiveness.

Behavior change techniques (BCTs) are observable, replicable, and irreducible components of interventions designed to alter or redirect causal processes that regulate behavior [12]. By specifying BCTs in interventions, researchers can identify the active ingredients in interventions, synthesize evidence, replicate interventions, and even optimize them [13]. Since the pandemic, remote self-management interventions for patients with hypertension based on mHealth apps have exploded, but there are great differences among studies in terms of intervention design, app contents, and study findings, which raise potential concerns about the generalizability of mHealth app–based interventions for hypertension. The presence of BCTs offers the possibility of optimizing the study design of the aforementioned studies and improving the effectiveness of the interventions. Previous literature reviews have described the efficacy of BCTs in mHealth for self-management of diabetes [14]. However, to the best of our knowledge, there is limited literature exploring the active ingredients of mHealth interventions for patients with hypertension. The status and role of BCTs in mHealth app self-management interventions for patients with hypertension are unclear.

### This Study

This study aimed to (1) evaluate the true effect of mHealth app self-management interventions on BP management in patients with hypertension, (2) comprehensively investigate the status of the use of BCTs in mHealth app interventions for patients with hypertension, and (3) further analyze what factors can influence the effect of interventions.

## Methods

### Overview

This systematic review and meta-analysis were carried out following the PRISMA (Preferred Reporting Items for Systematic Reviews and Meta-Analyses) guidelines (Multimedia Appendix 1) [15], and the review protocol was registered in PROSPERO (CRD42023483746).

### Search Strategy and Selection Criteria

With the assistance of a professional librarian, a systematic literature search was conducted in PubMed, Web of Science, Embase, American Psychological Association PsycINFO, CINAHL Plus, and Cochrane Central Register of Controlled Trials. In brief, we included randomized controlled trials (RCT) applying mHealth app self-management interventions among patients with hypertension and those published in English. Given that digital health apps started to become widely adopted in 2009, we set the search time from January 1, 2009, to October 15, 2023 [10]. We also performed a hand search of the reference lists of included studies and reviews related to the topic of this study to identify additional studies. The detailed search strategy is provided in Table S1 in Multimedia Appendix 2.

There were 5 eligibility criteria. The first was population: the study population was adults with a primary diagnosis of hypertension. We excluded studies involving minors, participants with pregnancy-related hypertension, and mixed patient populations without stratified results. The second eligibility criterion was intervention: the primary intervention was based on an mHealth app that can run on mobile or wearable devices such as smartphones, tablets, or smartwatches, and the duration of interventions was >4 weeks or 1 month. We excluded studies involving an intervention mainly based on phone calls, text messages, and website programs, as well as apps with only notification, self-monitoring, and counseling functions (interventions based on such apps may lack sufficient complexity and comprehensiveness to accurately reflect the true effect of mHealth app interventions on BP self-management). The third eligibility criterion was control: the study consisted of at least 1 intervention group and 1 control group that incorporated usual or standard care. Studies in which the control group applied the mHealth app were excluded. The fourth eligibility criterion was outcome: the primary outcomes were SBP, diastolic BP (DBP), or both; the secondary outcomes were clinical or self-reported indicators related to hypertension self-management, including but not restricted to the proportion of BP in control and medication adherence. the fifth eligibility criterion was study design and publication type: peer-reviewed RCTs, pilot studies, and cluster RCTs. We excluded nonrandomized, noncontrolled, and observational studies; case reports; systematic reviews; meta-analyses; conference abstracts; protocols; preprints; and studies without full text. In addition, we also excluded studies in which the intervention was not well described (eg, low-quality studies with overly simplistic descriptions of intervention methods that failed to identify intervention contents) to allow for valid coding of BCTs.

### Study Selection and Data Extraction

All identified studies were imported into EndNote X9 (Clarivate Analytics) software to remove duplicates and then imported into the Rayyan web platform (Rayyan Systems Inc) for eligibility review. The review process involved 2 rounds of screening: 2 reviewers (YZ and SJL) first screened the titles, abstracts, and keywords independently, and then the same 2 reviewers reviewed the full texts of the studies meeting the eligibility criteria to determine the list of included studies. Any disagreements in the review process were resolved through consultation and discussion with a senior reviewer (MHP).

Two reviewers (YZ and RQH) independently performed data extraction using a Microsoft Excel form developed following the guidelines in the Cochrane Handbook for Systematic Reviews of Interventions [16]. Any disagreements were resolved through rechecking the original research and discussion with another reviewer (MHP). The information recorded was (1) basic characteristics of publication: title, author, year, country of publication, and journal; (2) study details: study design, sample size, retention rate, intervention duration, detailed content of intervention and control group, and primary and secondary outcomes; (3) participants’ characteristics: age, sex ratio, education, ethnicity, and diagnostic criteria of hypertension; (4) outcomes: duration of follow-up, critical outcomes in BP changes, including the mean, SDs, SEs, and 95% CIs in baseline and follow-up, and validated measurements used for self-reported outcomes; and (5) theoretical foundations and BCTs applied in mHealth app interventions.

### Coding of BCTs

The coding of BCTs was performed according to the Taxonomy of BCTs (version 1) proposed by Michie et al [12], which summarized 93 BCTs into 16 categories and provided a standardized framework for identifying the BCTs used in behavior change interventions. In total, 2 reviewers (YZ and SJL) coded the BCTs independently after completing web-based training [17]. Evidence supporting BCT coding was from articles, supplementary materials, protocols, and secondary analysis publications. Any discrepancies in the coding process were resolved by discussion with another reviewer (MHP) until unanimity was achieved.

### Risk of Bias and Grade of Evidence Assessment

The revised Cochrane Risk of Bias (version 2) tools for RCTs and cluster RCTs were used to assess the risk of bias in (1) the randomization process, (2) deviations from the intended interventions, (3) missing outcome data, (4) measurement of the outcome, and (5) selection of the reported result of the included studies [18]. The Grading of Recommendation, Assessment, Development, and Evaluation criteria were used to assess the quality of the overall evidence in (1) risk of bias, (2) inconsistency, (3) indirectness, (4) imprecision, and (5) publication bias, and the quality of evidence could be classified as high, moderate, low, or very low [19]. In total, 2 reviewers (YZ and RQH) reviewed and rated the studies independently, and any disagreements were resolved via discussion with another reviewer (MHP).

### Data Synthesis and Analysis

A meta-analysis was performed to evaluate the pooled effect size of an mHealth app intervention on BP reduction. Given the potential bias caused by differences in BP levels between the intervention and control groups at baseline, we decided to include data on within-group changes in the mean and SD values of SBP and DBP in each group at follow-up in the analyses.

For studies in which the data were not available from articles, supplementary materials, and secondary analysis publications, the researcher attempted to contact the corresponding authors for necessary data. For studies that only reported SE or 95% CI or did not report the within-group changes in mean and SD values for BP, the researcher transformed the SE and 95% CI data and estimated the SD values according to the Cochrane handbook [16]. Studies in which analytical data were unavailable based on the aforementioned methods were excluded from the analysis.

Due to the considerable heterogeneity between the included studies, the random-effects model was considered appropriate to synthesize the effect sizes and SDs [20]. Heterogeneity was quantified using the Cochran *Q* test and Higgins *I*^2^ statistics, and the *I*^2^ values <25%, 25% to 75%, and >75% were considered low, medium, and high heterogeneity, respectively [21]. The bias of publication was evaluated by using the Egger test and visualization of the funnel plot.

Subgroup analyses were conducted to explore the impact of intervention and study design factors (such as the risk factors of hypertension that the mHealth app intervention addressed, the presence of a theoretical foundation, and intervention duration) and the number of BCTs on the effect size of an mHealth app intervention on BP levels and to evaluate the possible sources of heterogeneity. Review Manager software (version 5.4.1) was used to perform meta- and subgroup analyses, and Stata software (version 15.1; StataCorp) was applied to draw the funnel plot.

## Results

### Literature Screening

**Figure 1:**
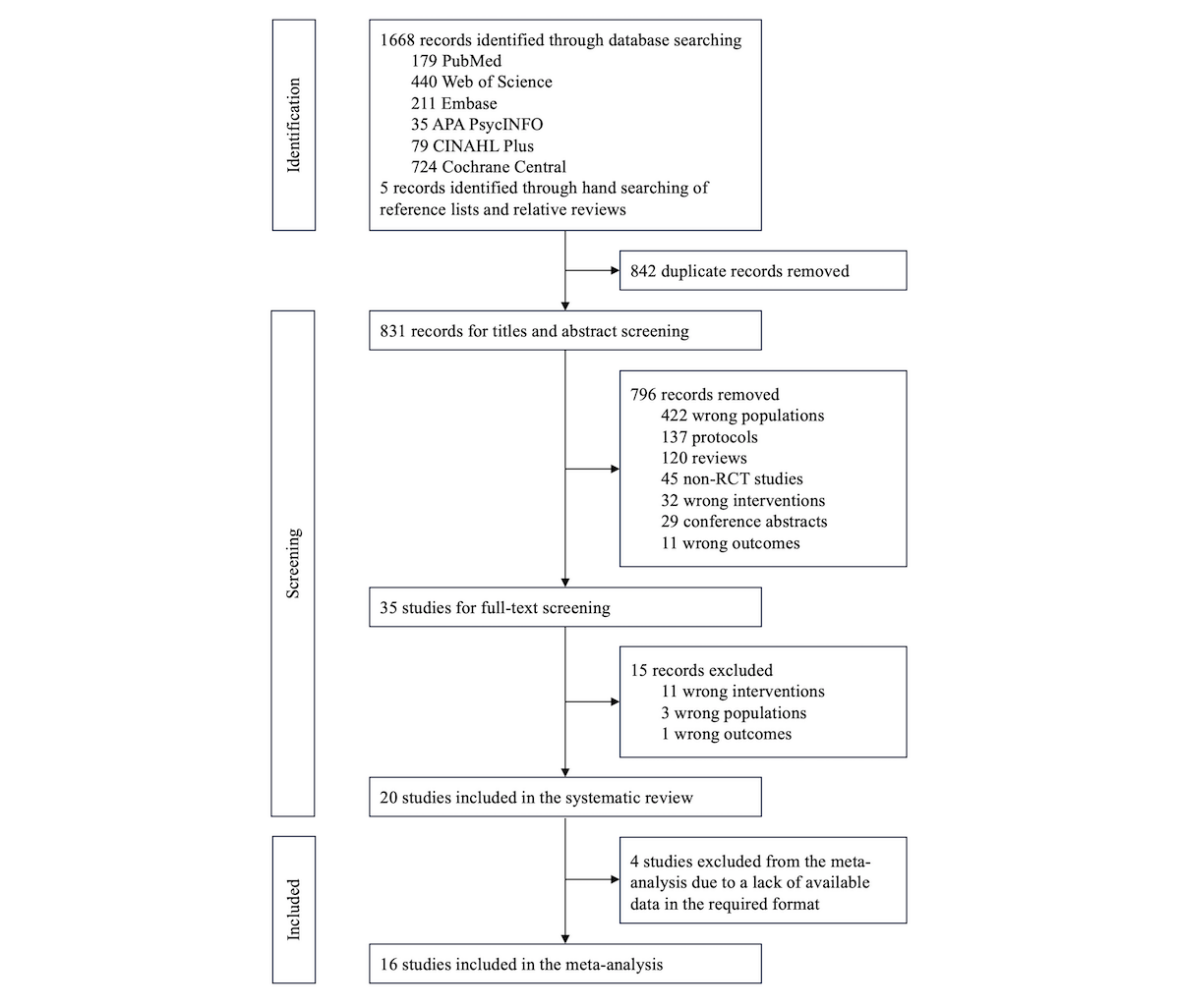
PRISMA (Preferred Reporting Items for Systematic Reviews and Meta-Analyses) flow diagram of the study selection process. RCT: randomized controlled trial.

The process of study identification and screening is outlined in Figure 1. A total of 1668 records were extracted from the initial literature search, and 5 records were identified from hand searching. After removing duplicates, 831 (49.67%) records were screened for titles and abstracts. Overall, 35 (4.2%) full-text studies were assessed for eligibility based on the inclusion and exclusion criteria. Finally, 20 (2.4%) studies were considered eligible and included in the systematic review, and 16 (1.9%) studies with available data were included in the meta-analysis.

### Study Characteristics

The characteristics of the included studies are presented in Table 1. A total of 20 studies were conducted in 9 countries, including China (6/20, 30%) [22-27], the United States (6/20, 30%) [28-33], Iran (2/20, 10%) [34,35], the United Kingdom (1/20, 5%) [36], Spain (1/20, 5%) [37], Germany (1/20, 5%) [38], Japan (1/20, 5%) [39], Palestine (1/20, 5%) [40], and Jordan (1/20, 5%) [41]. All studies were published after 2017, especially between 2020 and 2023. Of the 20 studies, 16 (80%) studies [22-24,26-28,30-36,39-41] used a parallel group design and 4 (20%) studies [25,29,37,38] used a cluster design. In total, 2 (10%) studies [33,36] addressed salt intake reduction and 2 (10%) studies [29,30] focused on medication management. In total, 2 (10%) studies [23,32] were conducted with ethnic minority groups and underserved and vulnerable populations. The number of participants in each study ranged from 30 to 636, and the duration of intervention ranged from 1.5 to 12 months. A total of 4168 participants were enrolled in the studies, with a retention rate of 85.2% (3551/4168).

**Table 1:** Characteristics of included studies (N=20).

Study, year	Country	Study design	Intervention content	Behavior change theory	Duration (months)	Retention, n/N (%)	Primary outcomes	Secondary outcomes	Conclusion
Abu-El-Noor et al [40], 2021	Palestine	RCT^a^	A smartphone app that included reminders for taking medication, follow-up appointments, educational information about hypertension management, and records of BP^b^ readings	—^c^	3	191/218 (87.6)	MAd (measured by Hill-Bone CHBPTSe) improved from 15.64 to 11.73 (intervention) and 15.92 to 13.98 (control; *P*<.001)	Hill-Bone CHBPTS improved from 30.82 to 23.40 (intervention) and 31.10 to 27.38 (control; *P*<.001) Diet adherence improved from 10.99 to 8.36 (intervention) and 10.90 to 9.65 (control; *P*=.001) Appointment adherence improved from 4.24 to 3.30 (intervention) and 4.28 to 3.76 (control)	Effective for adherence
Alsaqer and Bebis [41], 2022	Jordan	RCT	4 smartphone apps that encouraged self-monitoring of BP and record readings, adherence to medication, deep breathing exercises, and walking and counting steps daily; education for hypertension self-care; a public health nursing intervention that included telephone follow-up	—	3	74/80 (92)	ΔSBPf: –14 mm Hg (intervention) and –7.75 mm Hg (control; *P*=.001) ΔDBPg: –2.65 mm Hg (intervention) and –0.38 mm Hg (control; *P*=.14)	SC-HIh—maintenance improved from 37.06 to 67.01 (intervention) and 33.93 to 44.52 (control; *P*=.001) SC-HI—monitoring improved by 55.29 to 73.04 (intervention) and 52.70 to 54.59 (control; *P*=.001) SC-HI—confidence improved from 41.79 to 82.06 (intervention) and 40.12 to 40.85 (control; *P*=.001) Changes in all dimensions in SF-36i were significant after the intervention	Effective for SBP but not DBP and self-care
Bozorgi et al [34], 2021	Iran	RCT	A smartphone app that included BP records, reminders for drug consumption, visit date and BP measurement, a healthy diet and weight loss plans, disease knowledge, motivational and supportive programs for smoking cessation, critical BP alarm, customized messages about adherence to treatment, and usual treatment	—	2	118/120 (98.3)	MA (measured by the 14-item Hill-Bone Scale) improved from 58.5 to 65.1 (intervention) and 59.1 to 59.7 (control)	ΔMAPj: –12.6 mm Hg (intervention) and –16 mm Hg (control) Adherence to a low-salt diet improved from 14.3 to 18.4 (intervention) and 15.8 to 17.3 (control) Adherence to a low-fat diet improved from 16.0 to 17.86 (intervention) and 15.8 to 17.13 (control) ΔVPAk: 32.8 min/wk (intervention) and –21.8 min/wk (control) ΔMPAl: 91.5 min/wk (intervention) and 41.6 min/wk (control)	Effective for adherence and PA^m^
Chandler et al [28], 2019	United States	Small-scale efficacy RCT	The Smartphone Med Adherence Stops Hypertension intervention that included a smartphone app, global systems for a mobile electronic medication tray, and a Bluetooth-enabled BP device	Self-determination theory	9	54/56 (96)	ΔSBP: –30.5 mm Hg (intervention) and –5 mm Hg (control) ΔDBP: –12.6 mm Hg (intervention) and –5.2 mm Hg (control) The percentage of participants with controlled SBP (<140 mm Hg) improved from 0% to 92.3% (intervention) and 0% to 27.8% (control; *P*=.001)	MA (measured by MMASn) improved from 6.83 to 9.81 (intervention) and decreased from 6.99 to 6.84 (control; *P*<.001)	Effective for BP and MA
Dorsch et al [33], 2020	United States	Single-center, prospective pilot RCT	The LowSalt4Life mobile app that included just-in-time tailored messages that promote behavioral changes when the participant entered stores and restaurants, and easy scan and search for the foods at stores and restaurants to find options containing lower sodium content	Theory planned behavior; self-regulation theory	2	48/50 (96)	ΔSBP: –7.5 mm Hg (intervention) and –0.7 mm Hg (control; *P*=.12)	ΔKawasaki estimated 24-h urinary excretion of sodium –462 mg (intervention) and 381 mg (control; *P*=.03) Δ24-h urinary excretion of sodium –637 mg (intervention) and –322 mg (control; *P*=.47)	Effective for salt intake
Frias et al [29], 2017	United States	Prospective, cluster, pilot RCT	The DMO^o^ intervention included a smartphone app, medicines coencapsulated with an ingestible sensor, an adhesive wearable sensor patch, and a provider web portal and education and counseling from investigators	—	3	105/109 (96.3)	ΔSBP (in 4 weeks): –21.8 mm Hg (intervention) and –12.7 mm Hg (control)	ΔSBP (in 12 weeks): –20.9 mm Hg (intervention) and –15.2 mm Hg (control) ΔDBP (in 4 weeks): –9.0 mm Hg (intervention) and –5.9 mm Hg (control) ΔDBP (in 12 weeks): –8.6 mm Hg (intervention) and –5.8 mm Hg (control) The percentage of participants with controlled BP (<140/90 mm Hg; in 4 weeks): 81.2% (intervention) and 33.3% (control); in 12 weeks: 80.0% (intervention) and 51.7% (control) ΔGlycated hemoglobin A1c (in 12 weeks): –0.19 mmol/L (intervention) and +0.26 mmol/L (control) The overall MA when using DMO was ≥80%	Effective for BP
Gong et al [27], 2020	China	Multicenter RCT	A smartphone app that provided reminders of drug dose and BP measurement, and scientific information and suggestions about hypertension	—	6	443/480 (92.3)	ΔSBP: –8.99 mm Hg (intervention) and –5.92 mm Hg (control) ΔDBP: –7.04 mm Hg (intervention) and –4.14 mm Hg (control) The percentage of participants with controlled BP (<140/90 mm Hg) improved from 39% to 77% (intervention) and 39% to 67% (control; *P*=.01)	MA (measured by MMAS) was 55% (low), 42% (medium), and 3% (high) in the intervention group and 68% (low), 30% (medium), and 2% (high) in the control group (*P*=.004)	Effective for BP and MA
Kario et al [39], 2021	Japan	Multicenter pilot RCT	A smartphone app that included a personalized lifestyle‐modification program for lowering BP and standard lifestyle modification	—	6	140/146 (95.9)	ΔSBP (24-h ABPMp at 24 weeks): –0.47 mm Hg (intervention) and –0.042 mm Hg (control; *P*=.78) ΔDBP (24-h ABPM at 24 weeks): –1.3 mm Hg (intervention) and –0.2 mm Hg (control; *P*=.39)	ΔSBP (24-h ABPM at 16 weeks): 0.096 mm Hg (intervention) and –0.29 mm Hg (control; *P*=.88) ΔSBP (home BP at 16 weeks): –4.1 mm Hg (intervention) and –0.96 mm Hg (control; *P*=.06) ΔSBP (home BP self-monitoring at 24 weeks): –5.2 mm Hg (intervention) and –2.0 mm Hg (control; *P*=.07) ΔDBP (nighttime ABPM at 24 weeks): –3.2 mm Hg (intervention) and –0.042 mm Hg (control; *P*=.04) There were no significant changes in body weight, BMI, or waist circumference	Not effective for BP
Leupold et al [38], 2023	Germany	Prospective cluster RCT	The PIA app that included transmission of BP measurements, graphic display of BP over time with an individual target range, a medication plan, ordering of prescription refills, video education, and links to BP-related information	—	12	525/636 (82.5)	ΔSBP: –21.1 mm Hg (intervention) and –15.5 mm Hg (control; *P*<.001) ΔDBP: –11.3 mm Hg (intervention) and –8.2 mm Hg (control; *P*=.40) The percentage of participants with controlled BP (<140/90 mm Hg) was 62.6% (intervention) and 44.6% (control; *P*<.001)	—	Effective for BP
Li et al [25], 2019	China	Cluster RCT	Self-management intervention based on WeChat and included health education, health promotion, group chat, and BP monitoring	Self-efficacy theory	6	243/462 (52.6)	ΔSBP: –5.5 mm Hg (intervention) and 1.6 mm Hg (control; *P*<.001) ΔDBP: –1.3 mm Hg (intervention) and 2.1 mm Hg (control; *P*=.01) The percentage of participants with controlled BP (<140/90 mm Hg) improved from 60.9% to 83.6% (intervention) and decreased from 69.2% to 63.6% (control; *P*<.001)	ΔScore of hypertension: knowledge 2.3 (intervention) and 0.8 (control) ΔScore of self-efficacy 0.8 (intervention) and –0.6 (control) ΔScore of self-management: 7.3 (intervention) and –1.4 (control; *P*<.001) ΔScore of social support: 0.4 (intervention) and 0.7 (control)	Effective for BP and self-management
Ma et al [26], 2022	China	RCT	A smartphone app that included health education, individual self-care planning, daily records, and an automated weekly health report and nurse-led individual education and consultation sessions	—	3	191/210 (91)	ΔSBP: –11.63 mm Hg (intervention) and –1.01 mm Hg (control; *P*<.001) ΔDBP: –5.53 mm Hg (intervention) and 1.69 mm Hg (control; *P*<.001) The percentage of participants with controlled BP (<140/90 mm Hg) improved from 14.3% to 31.43% (intervention) and 7.6% to 8.57% (control; *P*=.003)	ΔWeight: –1.16 kg (intervention) and –0.03 kg (control) ΔBMI: –0.50 kg/m2 (intervention) and –0.09 kg/m2 (control) ΔWCq: –3.02 cm (intervention) and 0.82 cm (control) Self-care behavior (measured by HBP-HCPr) improved from 53.11 to 61.50 (intervention) and 54.82 to 54.94 (control); self-care motivation (measured by HBP-HCP) improved from 53.79 to 60.89 (intervention) and 55.02 to 55.27 (control); self-care–self-efficacy (measured by HBP-HCP) improved from 55.53 to 63.32 (intervention) and 55.60 to 56.13 (control)	Effective for BP and self-care
Márquez Contreras et al [37], 2018	Spain	Cluster RCT	A smartphone app that included personal data records, recommendations for target BP, physician’s medication advice, reminder alarms, calendar of appointments or events, and a record of BP measurement results	—	12	148/154 (96.1)	ΔSBP: –2.5 mm Hg (intervention) and –0.07 mm Hg (control; *P*<.001) ΔDBP: –3.14 mm Hg (intervention) and –0.5 mm Hg (control; *P*<.001)	Pharmacological therapeutic adherence was significantly improved after the intervention	Effective for BP and MA
Morawski et al [30], 2018	United States	RCT	The Medisafe mobile app that included reminder alerts of medication, adherence reports, tracks of BP, and optional peer support	—	3	411/412 (99.8)	ΔSBP: –10.6 mm Hg (intervention) and –10.1 mm Hg (control; *P*=.78) MA (measured by MMAS-8) improved from 6.0 to 6.3 (intervention) and maintained 5.7 (control; *P*=.001)	The percentage of participants with controlled BP (<140/90 mm Hg) improved from 0% to 35.8% (intervention) and 0% to 37.9% (control; *P*=.69)	Effective for MA but not SBP
Najafi Ghezeljeh et al [35], 2018	Iran	RCT	A Telegram group that included self-management education and contact and communication	—	1.5	50/50 (100)	Self-management behavior (measured by HSMBQs) improved from 2.23 to 3.73 (intervention) and decreased from 1.84 to 1.83 (control)	—	Effective for self-management
Persell et al [31], 2020	United States	RCT	Home BP self-monitoring and hypertension personal control program that included medication reminders, hypertension education, DASH^t^ diet encouragements, BP measuring reminders, coaching about PA, sleep tracks, stress management education, record reminders, and customized communication	Cognitive behavioral therapy	6	297/333 (88.2)	ΔSBP: –8.3 mm Hg (intervention) and –6.8 mm Hg (control; *P*=.16) ΔDBP: –4.3 mm Hg (intervention) and –3.6 mm Hg (control; *P*=.61) The percentage of participants with controlled BP (<140/90 mm Hg) improved from 36% to 72% (intervention) and 41% to 78% (control; *P*=.66)	Self-confidence in controlling BP significantly improved after the intervention (*P*<.001)	Not effective for BP
Payne Riches et al [36], 2021	United Kingdom	RCT	The SaltSwap mobile app that included brief advice on encouraging individuals to swap to lower-salt alternatives, buying fewer high-salt foods, and using less salt when cooking and face-to-face behavioral advice and support provided by a health care professional	Behavior change wheel	1.5	45/47 (96)	ΔSBP: –1.0 mm Hg (intervention) and –1.1 mm Hg (control; *P*=.82) ΔDBP: –1.0 mm Hg (intervention) and 2.3 mm Hg (control; *P*=.23)	Salt intake: –0.2 g/d (intervention) and –1.0 g/d (control; *P*=.68); purchased salt: –0.0 g/100 g (intervention) and –0.1 g/100 g (control; *P*=.16)	Not effective for BP and salt intake
Sun et al [24], 2020	China	RCT	3 WeChat groups (according to cardiovascular risk factors) that included health education, health behavior promotion, group chats, and BP monitoring	—	3	117/120 (98)	ΔSBP: –10.92 mm Hg (intervention) and –3.43 mm Hg (control; *P*<.001) ΔDBP: –5.68 mm Hg (intervention) and –2.23 mm Hg (control; *P*=.07)	Changes in TCu and LDL-Cv were significant after the intervention MA (measured by MMAS-8) improved from 2.28 to 3.30 (intervention) and 2.35 to 2.38 (control) Self-management behavior (measured by HPSMBRSw) improved from 72.27 to 74.57 (intervention) and 70.82 to 70.85 (control; *P*<.001) ΔBMI: –0.49 kg/m2 (intervention; *P*<.001) and –0.06 kg/m2 (control)	Effective for BP and MA
Zha et al [32], 2020	United States	Pilot RCT	The iHealth MyVitals mobile app included tracks and analysis of key health measurements and instant feedback, helping users self-monitor and manage BP, and standard hypertension management	—	6	25/30 (83)	ΔSBP: –8.39 mm Hg (intervention) and –4.79 mm Hg (control; *P*=.01) ΔDBP: –2.76 mm Hg (intervention) and –2.2 mm Hg (control)	MA self-efficacy (measured by MASESx) improved from 64.85 to 69.17 (intervention) and decreased from 64.75 to 61.00 (control; *P*=.06)	Effective for BP and self-management
Zhang et al [22], 2022	China	RCT	A smartphone app that included reminder alerts, access to historical data, and real-time chat and wearable device that can trace steps, heart rate, BP, and sleeping hours	—	6	192/307 (62.5)	ΔSBP: 3.2 mm Hg (intervention) and 6.4 mm Hg (control) ΔDBP: 2.7 mm Hg (intervention) and 5.4 mm Hg (control)	ΔBMI: 0.4 kg/m2 (intervention) and 0.6 kg/m2 (control) ΔWeight: 1.1 kg (intervention) and 1.5 kg (control)	Effective for BP
Zhang et al [23], 2023	China	RCT	A smartphone app that included reminder alerts, adherence reports, medical instruction, and optional family support and monitoring a wearable device (model unknown)	Theory of planned behavior	3	134/148 (90.5)	ΔSBP: –8.52 mm Hg (intervention) and –1.25 mm Hg (control; *P*=.01) ΔDBP: –0.42 mm Hg (intervention) and –0.01 mm Hg (control)	ΔWC: –2.14 cm (intervention) and –0.25 cm (control) ΔHCy: –0.30 cm (intervention) and –0.01 cm (control; *P*=.08) Self-efficacy improved: 12.89 (intervention) and 5.43 (control) Hypertension compliance improved: 7.35 (intervention) and 3.01 (control) Physical health improved: 12.21 (intervention) and 1.54 (control) Mental health improved: 13.17 (intervention) and 2.55 (control)	Effective for SBP but not DBP

^a^RCT: randomized controlled trial.

^b^BP: blood pressure.

^c^Not applicable.

^d^MA: medication adherence.

^e^CHBPTS: Compliance to High Blood Pressure Therapy Scale.

^f^SBP: systolic blood pressure.

^g^DBP: diastolic blood pressure.

^h^SC-HI: Self-Care of Hypertension Inventory.

^i^SF-36: 36-Item Short Form Survey.

^j^MAP: mean arterial pressure.

^k^VPA: vigorous physical activity.

^l^MPA: moderate physical activity.

^m^PA: physical activity.

^n^MMAS: Morisky Medication Adherence Scale.

^o^DMO: digital medicine offering.

^p^ABPM: ambulatory blood pressure monitoring.

^q^WC: waist circumference.

^r^HBP-HCP: Hypertension Self-care Profile.

^s^HSMBQ: Hypertension Self-Management Behavior Questionnaire.

^t^DASH: Dietary Approaches to Stop Hypertension.

^u^TC: total cholesterol.

^v^LDL-C: low-density lipoprotein cholesterol.

^w^HPSMBRS: Hypertension Patients Self-Management Behavior Rating Scale.

^x^MASES: Medication Adherence Self-Efficacy Scale.

^y^HC: hip circumference.

### Risk of Bias and Grade of Evidence

**Figure 2:**
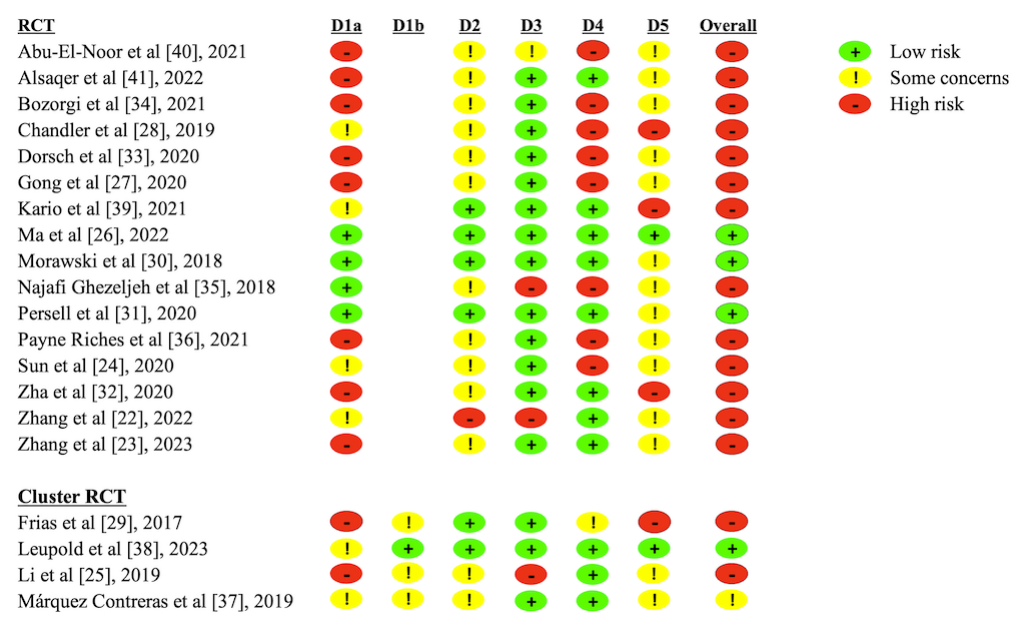
Risk of bias assessment of the included studies [22-41] using the Cochrane Risk of Bias (version 2) tool. RCT: randomized controlled trial.

Of the 20 included studies, 15 (75%) [22-25,27-29,32-36,39-41] were judged as high risk of bias, 1 (5%) [37] was judged as having some concerns, and 4 (20%) [26,30,31,38] were judged as low risk of bias (Figure 2 [22-41]). Overall, the risk of bias primarily existed in the randomization process and measurement of the outcome. In terms of the randomization process, 50% (10/20) of the studies [23,25,27,29,32-34,36,40,41] were rated as high risk, primarily because of not following the principle of, or not providing details about, randomization and allocation concealment. Regarding the measurement of the outcome, 40% (8/20) of the studies [24,27,28,33-36,40] were assessed as high risk mainly due to the use of patients’ self-reported subjective outcomes and the lack of disclosing whether the outcome measurers were aware of the intervention patients received. In addition, the lack of blinding among patients and between patients and investigators during the intervention process (although often difficult to achieve in mHealth interventions), high dropout rates and not conducting intention-to-treat analyses, and selective disclosure of predetermined outcome data were also important sources of risk of bias. The result of the Egger test (*P*=.16) and visualization of the funnel plot (Figure S1 in Multimedia Appendix 2) indicated that there was no significant risk of publication bias in the included studies.

The results of the Grading of Recommendation, Assessment, Development, and Evaluation assessment are presented in Table 2. In terms of SBP, the quality of evidence was low, and for DBP, the quality of evidence was moderate.

**Table 2:** Grading of Recommendation, Assessment, Development, and Evaluation assessment.

Blood pressure	Studies, n	Quality assessment						Patients (E^a^/C^b^)		Effect, mean difference (95% CI)		Overall certainty of evidence
		Risk of bias	Inconsistency	Indirectness	Imprecision	Publication bias						
SBP^c^	16	Serious^d^	Serious^e^	Not serious	Not serious	None	1568/1516		–5.78 (–7.97 to 3.59)		Low	
DBP^f^	15	Serious^d^	Not serious	Not serious	Not serious	None	1359/1316		–3.28 (–4.39 to –2.17)		Moderate	

^a^E: experimental group.

^b^C: control group.

^c^SBP: systolic blood pressure.

^d^The included studies were judged as have a high risk of bias overall. Some of the studies had evident risks in the randomization process and measurement of the outcome.

^e^Cochran *Q* test and Higgins *I^2^* suggested significant heterogeneity between studies.

^f^DBP: diastolic blood pressure.

### Behavior Change Theories and Techniques

Table 1 and Table S2 in Multimedia Appendix 2 summarize the behavior change theories and BCTs used in the included studies. Of the 20 studies, 6 (30%) [23,25,28,31,33,36] reported 7 behavior change theories in their interventions and the remaining 14 (70%) did not include a theoretical foundation. Theories involved in the interventions included self-determination theory [28], theory of planned behavior [23,33], self-regulation theory [33], self-efficacy theory [25], cognitive behavioral theory [31], and behavior change wheel [36]. Of the 6 studies, 1 (17%) used 2 theories [33] and the remaining 5 (83%) were based on a single theory.

A total of 21 different BCTs from 12 BCT categories were reported in the included 20 studies. The mean number of BCTs was 8.7 (SD 3.8), with a range of 2 to 17, accounting for 2% (2/93) to 18% (17/93) of the total 93 BCTs. The frequently used BCT clusters (used in ≥10 studies) were *goals and planning*, *feedback and monitoring*, *shaping knowledge*, *associations*, *comparison of outcomes*, and *regulation*. The most common BCTs were *self-monitoring of outcomes of behavior* (17/20, 85%), *feedback on outcomes of behavior* (15/20, 75%), *instruction on how to perform the behavior* (15/20, 75%), *pharmacological support* (15/20, 75%), *biofeedback* (14/20, 70%), *prompts/cues* (13/20, 65%), *credible source* (13/20, 65%), and *action planning* (11/20, 55%). There were 4 BCTs used only in 10% (2/20) of the studies: *problem solving*, *information about health consequences*, *behavior substitution*, and *conserving mental resources*.

### Effects of an mHealth App Intervention on BP

#### Effect of an mHealth App Intervention on SBP

**Figure 3:**
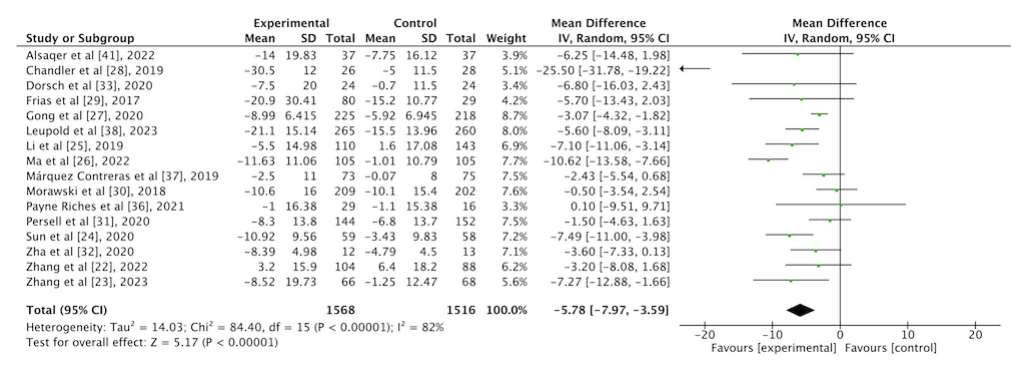
Forest plot of the overall effect of a mobile health app intervention on systolic blood pressure.

In total, 16 (80%) of the 20 studies reported the effects of an mHealth app intervention on SBP. A total of 1568 participants from the intervention group and 1516 participants from the control group were included in the meta-analysis. As presented in Figure 3, the mHealth app intervention resulted in a –5.78 mm Hg (95% CI –7.97 mm Hg to –3.59 mm Hg) reduction in SBP. The heterogeneity was significant (*I*^2^=82%; *P*<.001) between the studies.

#### Effect of an mHealth App Intervention on DBP

**Figure 4:**
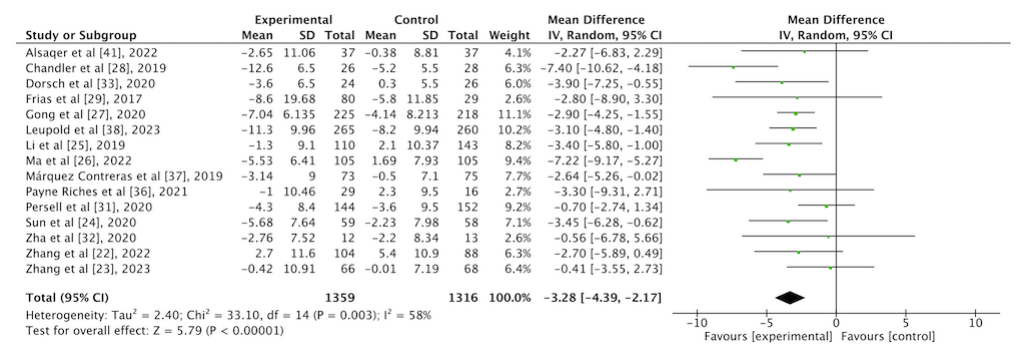
Forest plot of the overall effect of a mobile health app intervention on diastolic blood pressure.

As shown in Figure 4, in total, 15 (75%) of the 20 studies reporting the effect of an mHealth app intervention on DBP including 1359 participants from the intervention group and 1316 participants from the control group were included in the analysis. Overall, the mHealth app intervention resulted in a –3.28 mm Hg (95% CI –4.39 mm Hg to –2.17 mm Hg) reduction in DBP with a medium heterogeneity (*I*^2^*=*58%; *P*=.003).

### Subgroup Analyses

Subgroup analyses were subsequently conducted to explore the impact of factors, such as the risk factors of hypertension that mHealth app interventions addressed (addressing a single risk factor vs addressing multiple risk factors), the presence of a theoretical foundation (with behavior change theories vs without behavior change theories), duration of the intervention (<6 vs ≥6 months), and the number of BCTs (≥11 vs<11), on the effect size of an mHealth app intervention on SBP.

### Factors Addressed in an mHealth App Intervention

Of the 16 studies that reported the effects of an mHealth app intervention on SBP, 12 (75%) studies [22-28,31,32,37,38,41] carried out interventions (eg, education of disease; BP monitoring and alerts; instructions on diet, salt intake, exercise, sleep, and stress management; and medication reminders or management) addressing multiple physiological and behavioral risk factors of hypertension, and the remaining 4 (25%) studies [29,30,33,36] carried out interventions (eg, reduction of salt intake or medication adherence management) addressing a single physiological or behavioral risk factor. As illustrated in Figure S2 in Multimedia Appendix 2, compared with the interventions addressing a single risk factor of hypertension (–1.54 mm Hg, 95% CI –4.15 mm Hg to 1.06 mm Hg), interventions addressing multiple risk factors of hypertension resulted in a better reduction in SBP (–6.50 mm Hg, 95% CI –9.00 mm Hg to –3.99 mm Hg) and the difference between subgroups was statistically significant (*P*=.007). However, the heterogeneity between studies addressing multiple risk factors remained significant (*I*^2^=86%; *P*<.001).

Given the obvious heterogeneity in studies with interventions addressing multiple physiological and behavioral risk factors of hypertension and that hypertension is a disease associated with multiple risk factors, the effect of mHealth apps addressing a single physiological or behavioral risk factor may differ from the effect of those addressing multiple risk factors. Therefore, subsequent subgroup analyses were conducted based on the 12 studies addressing multiple physiological and behavioral risk factors of hypertension.

### Use of Behavior Change Theory

Of the 12 studies, 5 (42%) studies [23,25,26,28,31] used behavior change theory and 7 (58%) studies [22,24,27,32,37,38,41] did not disclose the theoretical foundation. As presented in Figure S3 in Multimedia Appendix 2, although the difference between subgroups was not statistically significant (*P*=.07), interventions with behavior change theory resulted in a –10.06 mm Hg (95% CI –16.42 mm Hg to –3.70 mm Hg) reduction in SBP, which is better than without behavior change theory with a –4.13 mm Hg (95% CI –5.50 mm Hg to –2.75 mm Hg) reduction in SBP. The heterogeneity among studies with behavior change theory was considerable (*I*^2^=92%; *P*<.001).

### Duration of Intervention

In the 12 studies, the duration of intervention ranged from 3 to 12 months, with a mean duration of 6.25 months. Among them, 4 (33%) studies [23,24,26,41] had a duration of 3 months, and the other 8 (67%) studies [22,25,27,28,31,32,37,38] had a duration of at least 6 months. The results of the subgroup analysis showed that the effect of the 3-month intervention duration (–8.87 mm Hg, 95% CI –10.90 mm Hg to –6.83 mm Hg) on reducing SBP was superior to those with a longer duration (–5.76 mm Hg, 95% CI –8.74 mm Hg to –2.77 mm Hg; *P*=.09). Apparent heterogeneity persisted between studies with interventions lasting longer than 6 months (*I*^2^=86%; *P*<.001; Figure S4 in Multimedia Appendix 2).

### Use of BCTs

A total of 19 different BCTs were used in 12 mHealth app interventions, with the number ranging from 2 to 12 and an average of 10.58 BCTs per intervention. In total, 7 (58%) of the 12 studies [23-26,28,38,41] used at least 11 BCTs. As shown in Figure S5 in Multimedia Appendix 2, interventions using at least 11 BCTs (–9.68 mm Hg, 95% CI –13.49 mm Hg to –5.87 mm Hg) had a statistically significant effect on the reduction in SBP compared to studies using fewer BCTs (–2.88 mm Hg, 95% CI –3.90 mm Hg to –1.86 mm Hg; *P*<.001).

## Discussion

### Principal Findings

In this study, we conducted a systematic review of 20 studies about mHealth app interventions for hypertension and performed a meta-analysis of 16 (80%) of the studies. The results indicated that mHealth app interventions resulted in a significant reduction in SBP (*P*<.001) and DBP (*P*<.001) compared to usual care, and the effect size was influenced by factors of intervention design (eg, presence of a theoretical foundation, intervention duration, and number of BCTs) and app contents (eg, the risk factors of the hypertension app addressed). Our study further demonstrates the effectiveness of mHealth app interventions in hypertension self-management and, for the first time, provides an interpretation of the active ingredients in such interventions from a BCT perspective. However, significant differences in intervention designs and the number and selection of BCTs across studies also indicated that these interventions may not be generalized in different social settings, and there is currently a lack of a unified guidance framework in mHealth interventions for hypertension.

Comprehensive intervention based on antihypertensive medications combined with lifestyle modifications is considered a standard strategy for the management of hypertension [42,43]. Nonpharmacological management is a multidimensional task that includes weight loss, the Dietary Approaches to Stop Hypertension diet, sodium reduction, potassium supplementation, increased physical activity, and reduction in tobacco and alcohol consumption [44]. As we guessed, due to covering more lifestyle modifications, mHealth apps addressing multiple physiological and behavioral risk factors of hypertension and offering more functions were more effective in lowering SBP. This finding was consistent with previous studies conducted in various populations [45-48]. Notably, the interventions in studies by Chandler et al [28] and Frias et al [29] resulted in more reduced SBP in absolute values compared to other mHealth app interventions. In those studies, the researchers applied medication trays with a series of reminder signals and ingestible sensors to enhance medication management, indicating that the strategies of enhanced reminders and tracking of medication behavior might be potential ways to improve medication management. Epidemiological evidence suggests that nonadherence to antihypertensive medications is as high as 27% to 40% globally, and there is still significant room for improvement in patients’ medication behavior [49]. Therefore, developing a multicomponent mHealth app for lifestyle modifications and conducting intensive intervention for medication behavior may be a viable direction for future mHealth interventions in hypertension.

Intervention duration is a key factor in intervention design, and it was noteworthy that, in our study, 20% (4/20) of the studies with an intervention duration of 3 months were more effective in reducing SBP than studies lasting ≥6 months. This finding was consistent with a previous study, in which Ma et al [50] found that the effect of a habit formation intervention on physical activity habits was better if the intervention duration was <12 weeks. Given that the contents of mHealth app interventions primarily involve lifestyle modifications and the formation of healthy living habits, this phenomenon seems to be partly explained by habit formation. The law of automaticity change in the formation of new habits indicates that habit strength usually reaches a peak of automaticity at approximately 12 weeks and gradually weakens over the following period [50,51]. In addition, of these 4 studies, 2 (50%) [24,26] involved educational curriculum programs that progressed over time. We suggested that, in addition to habit formation, the enhancement of intrinsic motivation for behavior change by planned sessions on disease, medications, and coping may also contribute to BP management. Therefore, how to promote the development of healthy living habits and maintain the habits and motivation of behavior change over a long period of the intervention will be an issue to consider in future study designs of similar interventions.

A theory is a set of interrelated concepts, definitions, and propositions that explain or predict events or situations by specifying relations among variables [52]. The existing view is that effective interventions used to promote healthy lifestyles and reduce risky behaviors are inseparable from the evidence of theories [53]. Similar to our findings, theory-based mHealth interventions have been found more effective in other chronic diseases and behavioral change studies [14,54]. A possible explanation is that, based on theories, researchers can identify causal factors associated with behavior change and the pathways through which behavior change occurs. Furthermore, this enhances treatment fidelity, allowing for a more comprehensive design of the protocol, early detection of errors and protocol deviations, and improvement in treatment retention, to maximize the effectiveness of interventions [43,55]. In our review, 6 studies disclosed the theories used, and the theory use rate was 30% (6/20). The lack of a theoretical foundation seems to have become a common phenomenon in current behavior change interventions [56,57]. We believe that this phenomenon needs urgent attention. With the development of mobile information technology, an increasing number of interventions are being based on mobile, smart, and wearable devices and are being transferred from health care institutions to patients’ homes. In the absence of face-to-face communication and strict supervision in telemedicine, holistic study designs based on theories will be particularly important to improve the effectiveness and generalization of the interventions. Future researchers can try to design intervention protocols based on multiple theories and integrate these theories to explore the best model for mHealth app interventions for hypertension.

Research evidence, including our study, has suggested that using more BCTs may be associated with better outcomes of behavior change [58,59]. However, some studies found that fewer techniques and the right combinations of techniques are more effective [60-62]. Indeed, due to the small effect of a single BCT, the fact that BCTs are often present in combinations in interventions, and the possibility of interactions between BCTs, determining which specific BCT, the combinations of BCTs, and the number of BCTs that are effective for a given behavior is a challenge [63]. Therefore, based on applying a certain number of BCTs and effective BCT combinations, customizing an intervention to the patient’s behavior change needs, including contents, duration, and delivery, may be another possible method to improve the effectiveness of the interventions.

This study also had some limitations. First, we intended to evaluate the effectiveness of mHealth app interventions on several subjective and objective outcomes including BP, medication adherence, self-efficacy, etc. However, due to the limited number of included studies and significant heterogeneity of subjective outcome measures, we ultimately only reported the result of BP management, which was inconsistent with the published protocol. In addition, although we attempted to explore the source of heterogeneity through subgroup analyses, heterogeneity remained significant in some subgroups, and because the included studies were all published after 2017, we also failed to explore the changes in the effectiveness of mHealth app interventions over time. It is recommended that future studies incorporate additional studies and conduct extensive subgroup analyses to further explore the impact of mHealth app interventions on self-management–related outcomes in patients with hypertension. Second, in this study, we only included app-based mHealth interventions and excluded studies based on phone calls, text messages, and website programs, which also contained BCTs, potentially leading to bias in the assessment of effective BCTs. Third, the evidence for BCT coding relies on information about intervention contents from available articles, supplementary materials, and secondary analysis publications, which were underreported or roughly outlined. Furthermore, despite the coding process being performed by 2 researchers independently with good consistency, BCT coding is inevitably susceptible to researchers’ subjective judgments. Therefore, the efficacy of BCTs for hypertension self-management still requires further validation in future studies. Finally, while our study coded BCTs and initially explored the effectiveness of the number of BCTs in SBP reduction, considering the wide variations of intervention contents and specific forms, frequencies, and intensities of BCTs across studies, we failed to further quantitatively investigate the effect of a single BCT or a combination of BCTs on BP reduction. Self-management of hypertension is a complex and multidimensional intervention, involving lifestyle modifications and medication management, and is affected by factors such as patients’ knowledge, intention, self-efficacy, and environment. It is foreseeable that more BCTs will be found effective in self-management of hypertension. Future research could further explore other BCTs and combinations of BCTs to provide references for the development of relevant mHealth interventions and apps.

### Conclusions

This systematic review and meta-analysis further confirmed the effectiveness of mHealth app self-management interventions for hypertension and identified the BCTs used in the interventions. Our study found that mHealth app interventions can lead to a reduction in SBP and DBP compared to usual care; factors related to the intervention and study design, such as the risk factors of hypertension the mHealth app intervention addressed, the presence of a theoretical foundation, intervention duration, and the number of BCTs, were associated with the effect sizes of BP reduction; and the most commonly used BCTs included *self-monitoring of outcomes of behavior*, *feedback on outcomes of behavior*, *instruction on how to perform the behavior*, and *pharmacological support*. On the basis of the findings of our study, future research can optimize the intervention designs and use more BCTs and BCT combinations to develop more effective mHealth apps and interventions for hypertension management.

## Appendix

Multimedia Appendix 1 PRISMA (Preferred Reporting Items for Systematic Reviews and Meta-Analyses) checklist.

Multimedia Appendix 2 Supplementary tables and figures.

## Abbreviations

BCT: behavior change technique
BP: blood pressure
DBP: diastolic blood pressure
mHealth: mobile health
PRISMA: Preferred Reporting Items for Systematic Reviews and Meta-Analyses
RCT: randomized controlled trial
SBP: systolic blood pressure

## Data Availability

The datasets used and analyzed during this study are available from the corresponding author on reasonable request.
