# Supplementary material for: Behavior Change Techniques Used in Self-Management Interventions Based on mHealth Apps for Adults With Hypertension: Systematic Review and Meta-Analysis of Randomized Controlled Trials
Source: J Med Internet Res. 2024 Oct 22;26:e54978. doi: 10.2196/54978 (PMC11538878; doi:10.2196/54978)
Supplement: Multimedia Appendix 2 [file jmir_v26i1e54978_app2.docx]

**Table S1. Detailed information of search strings.**

| PubMed | |
| --- | --- |
| #1 | (hypertension[Mesh]) OR (hypertension[Title/Abstract]) OR (hypertensive[Title/Abstract]) OR (blood pressure[Title/Abstract]) OR (SBP[Title/Abstract]) OR (DBP[Title/Abstract]) OR (elevated blood pressure) OR (high blood pressure[Title/Abstract]) |
| #2 | (telemedicine[Mesh]) OR (mhealth[Title/Abstract]) OR (m-health[Title/Abstract]) OR (mobile health[Title/Abstract]) OR (digital tool*[Title/Abstract]) OR (digital technology[Title/Abstract]) OR (smart phone*[Title/Abstract]) OR (smartphone*[Title/Abstract]) OR (mobile phone*[Title/Abstract]) OR (iphone[Title/Abstract]) OR (ios[Title/Abstract]) OR (android[Title/Abstract]) OR (apps[Title/Abstract]) OR (mobile application*[Title/Abstract]) OR (wearable[Title/Abstract]) OR (mobile device*[Title/Abstract]) |
|  |  |
|  |  |
|  |  |
|  |  |
| #3 | (self-management[Mesh]) OR (self-manag*[Title/Abstract]) OR (self manag*[Title/Abstract]) OR (self-car*[Title/Abstract]) OR (self car*[Title/Abstract]) OR (self-monitor*[Title/Abstract]) OR (self monitor*[Title/Abstract]) OR (self-regulat*[Title/Abstract]) OR (self regulat*[Title/Abstract]) OR (self-control*[Title/Abstract]) OR (self control*[Title/Abstract]) OR (disease-manag*[Title/Abstract]) OR (disease manag*[Title/Abstract]) |
|  |  |
|  |  |
|  |  |
|  |  |
|  |  |
|  |  |
|  |  |
| #4 | (clinical trial[Title/Abstract]) OR (randomized controlled trial[Title/Abstract]) |
| #5 | #1 AND #2 AND #3 AND #4 |
| Web of Science | |
| #1 | TS=(hypertension) OR (hypertensive) OR (blood pressure) OR (SBP) OR (DBP) OR (elevated blood pressure) OR (high blood pressure) |
|  |  |
|  |  |
| #2 | TS=(mhealth) OR (m-health) OR (mobile health) OR (digital tool*) OR (digital technology) OR (smart phone*) OR (smartphone*) OR (mobile phone*) OR (iphone) OR (ios) OR (android) OR (apps) OR (mobile application*) OR (wearable) OR (mobile device*) |
|  |  |
|  |  |
|  |  |
| #3 | TS=(self-manag*) OR (self manag*) OR (self-car*) OR (self car*) OR (self-monitor*) OR (self monitor*) OR (self-regulat*) OR (self regulat*) OR (self-control*) OR (self control*) OR (disease-manag*) OR (disease manag*) |
|  |  |
|  |  |
|  |  |
| #4 | TS=(clinical trial) OR (randomized controlled trial) OR (controlled clinical trial) |
|  |  |
| #5 | #1 AND #2 AND #3 AND #4 |
| Embase | |
| #1 | 'hypertension'/exp |
| #2 | 'hypertension':ab,ti OR 'hypertensive':ab,ti OR 'blood pressure':ab,ti OR 'sbp':ab,ti OR 'dbp':ab,ti OR 'elevated blood pressure':ab,ti OR 'high blood pressure':ab,ti |
| #3 | #1 OR #2 |
| #4 | 'telemedicine'/exp |
| #5 | 'mhealth':ab,ti OR 'm-health':ab,ti OR 'mobile health':ab,ti OR 'digital tool*':ab,ti OR 'digital technology':ab,ti OR 'smart phone*':ab,ti OR 'smartphone*':ab,ti OR 'mobile phone*':ab,ti OR 'iphone':ab,ti OR 'ios':ab,ti OR 'android':ab,ti OR 'apps':ab,ti OR 'mobile application*':ab,ti OR 'wearable':ab,ti OR 'mobile device*':ab,ti |
| #6 | #4 OR #5 |
| #7 | ‘self-management'/exp |
| #8 | ‘self-manag*':ab,ti OR 'self manag*':ab,ti OR 'self-car*':ab,ti OR 'self car*':ab,ti OR 'self-monitor*':ab,ti OR 'self monitor*':ab,ti OR 'self-regulat*':ab,ti OR 'self regulat*':ab,ti OR 'self-control*':ab,ti OR 'self control*':ab,ti OR 'disease-manag*':ab,ti OR 'disease manag*':ab,ti |
| #9 | #7 OR #8 |
| #10 | ‘clinical trial':ab,ti OR 'randomized controlled trial':ab,ti OR 'controlled clinical trial':ab,ti |
| #11 | #3 AND #6 AND #9 AND #10 |
| APA PsycInfo | |
| #1 | AB (hypertension OR hypertensive OR blood pressure OR SBP OR DBP OR elevated blood pressure OR high blood pressure) |
| #2 | AB (mhealth OR m-health OR mobile health OR digital tool* OR digital technology OR smart phone* OR smartphone* OR mobile phone* OR iphone OR ios OR android OR apps OR mobile application* OR wearable OR mobile device*) |
| #3 | AB (self-manag* OR self manag* OR self-car* OR self car* OR self-monitor* OR self monitor* OR self-regulat* OR self regulat* OR self-control* OR self control* OR disease-manag* OR disease manag*) |
| #4 | AB (clinical trial OR randomized controlled trial OR controlled clinical trial) |
| #5 | #1 AND #2 AND #3 AND #4 |
| CINHAL | |
| #1 | MH (hypertension) |
| #2 | AB (hypertension OR hypertensive OR blood pressure OR SBP OR DBP OR elevated blood pressure OR high blood pressure) |
| #3 | #1 OR #2 |
| #4 | MH (telemedicine) |
| #5 | AB (mhealth OR m-health OR mobile health OR digital tool* OR digital technology OR smart phone* OR smartphone* OR mobile phone* OR iphone OR ios OR android OR apps OR mobile application* OR wearable OR mobile device*) |
| #6 | #4 OR #5 |
| #7 | MH (self-Management) |
| #8 | AB (self-manag* OR self manag* OR self-car* OR self car* OR self-monitor* OR self monitor* OR self-regulat* OR self regulat* OR self-control* OR self control* OR disease-manag* OR disease manag*) |
| #9 | #7 OR #8 |
| #10 | AB (clinical trial OR randomized controlled trial OR controlled clinical trial) |
| #11 | #3 AND #6 AND #9 AND #10 |
| Cochrane Central Register of Controlled Trials | |
| #1 | (hypertension):ab,ti,kw OR (hypertensive):ab,ti,kw OR (blood pressure):ab,ti,kw OR (SBP):ab,ti,kw OR (DBP):ab,ti,kw OR (elevated blood pressure):ab,ti,kw OR (high blood pressure):ab,ti,kw |
| #2 | (mhealth):ab,ti,kw OR (m-health):ab,ti,kw OR (mobile health):ab,ti,kw OR (digital tool*):ab,ti,kw OR (digital technology):ab,ti,kw OR (smart phone*):ab,ti,kw OR (smartphone*):ab,ti,kw OR (mobile phone*):ab,ti,kw OR (iphone):ab,ti,kw OR (ios):ab,ti,kw OR (android):ab,ti,kw OR (apps):ab,ti,kw OR (mobile application*):ab,ti,kw OR (wearable):ab,ti,kw OR (mobile device*):ab,ti,kw |
| #3 | (self-manag*):ab,ti,kw OR (self manag*):ab,ti,kw OR (self-car*):ab,ti,kw OR (self car*):ab,ti,kw OR (self-monitor*):ab,ti,kw OR (self monitor*):ab,ti,kw OR (self-regulat*):ab,ti,kw OR (self regulat*):ab,ti,kw OR (self-control*):ab,ti,kw OR (self control*):ab,ti,kw OR (disease-manag*):ab,ti,kw OR (disease manag*):ab,ti,kw |
| #4 | #1 AND #2 AND #3 |

**Table S2. Behavior change techniques reported in the included studies (N=20).**

| Study | 1.1 Goal setting (behavior) | 1.2 Problem solving | 1.3 Goal setting (outcome) | 1.4 Action planning | 2.2 Feedback on behavior | 2.3 Self monitoring of behavior | 2.4 Self-monitoring of outcomes of behavior | 2.6 Biofeedback | 2.7 Feedback on outcomes of behavior | 3.1 Social support (unspecified) | 4.1 Instruction on how to perform the behavior | 5.1 Information about health consequences | 6.1 Demonstration of the behavior | 7.1 Prompts/cues | 8.2 Behavior substitution | 9.1 Credible source | 10.4 Social reward | 11.1 Pharmacological support | 11.2 Reduce negative emotions | 11.3 Conserving mental resources | 12.5 Adding objects to the environment |
| --- | --- | --- | --- | --- | --- | --- | --- | --- | --- | --- | --- | --- | --- | --- | --- | --- | --- | --- | --- | --- | --- |
| Abu-El-Noor et al [40], 2021 |  |  |  |  |  |  | ✓ |  |  |  | ✓ |  |  | ✓ |  | ✓ |  | ✓ |  |  |  |
| Alsaqer and Bebis [41], 2022 |  |  |  |  | ✓ | ✓ | ✓ | ✓ | ✓ | ✓ | ✓ |  |  | ✓ |  | ✓ |  | ✓ | ✓ |  |  |
| Bozorgi et al [34], 2021 |  |  |  | ✓ |  |  | ✓ |  |  | ✓ | ✓ |  |  | ✓ |  |  | ✓ | ✓ |  |  |  |
| Chandler et al [28], 2019 | ✓ |  | ✓ | ✓ |  |  | ✓ | ✓ | ✓ | ✓ | ✓ |  | ✓ | ✓ |  | ✓ |  | ✓ |  |  | ✓ |
| Dorsch et al [33], 2020 |  |  |  |  |  |  |  |  |  |  |  |  |  | ✓ | ✓ |  |  |  |  | ✓ |  |
| Frias et al [29], 2017 |  |  |  |  |  |  |  |  | ✓ |  | ✓ |  |  |  |  |  |  | ✓ |  |  | ✓ |
| Gong et al [27], 2020 |  |  |  | ✓ |  | ✓ | ✓ | ✓ |  |  | ✓ |  |  | ✓ |  | ✓ |  | ✓ |  |  |  |
| Kario et al [39], 2021 |  |  |  | ✓ |  |  | ✓ | ✓ | ✓ |  | ✓ |  | ✓ |  |  | ✓ |  |  |  |  |  |
| Leupold et al [38], 2023 |  |  | ✓ | ✓ |  | ✓ | ✓ | ✓ | ✓ | ✓ | ✓ |  | ✓ | ✓ |  | ✓ |  | ✓ |  |  |  |
| Li et al [25], 2019 | ✓ | ✓ | ✓ | ✓ |  | ✓ | ✓ | ✓ | ✓ | ✓ | ✓ |  |  |  |  | ✓ | ✓ | ✓ | ✓ |  |  |
| Ma et al [26], 2022 | ✓ |  | ✓ | ✓ | ✓ | ✓ | ✓ | ✓ | ✓ | ✓ | ✓ | ✓ | ✓ | ✓ |  | ✓ |  | ✓ | ✓ |  |  |
| Márquez Contreras et al [37], 2018 |  |  | ✓ | ✓ |  |  | ✓ | ✓ |  |  | ✓ |  |  | ✓ |  | ✓ |  | ✓ |  |  |  |
| Morawski et al [30], 2018 |  |  |  |  |  |  | ✓ | ✓ | ✓ | ✓ |  |  |  | ✓ |  |  |  | ✓ |  |  | ✓ |
| Najafi Ghezeljeh et al [35], 2018 |  |  |  |  |  |  | ✓ |  | ✓ |  | ✓ |  | ✓ |  |  |  |  | ✓ |  |  |  |
| Persell et al [31], 2020 |  |  |  |  | ✓ |  | ✓ | ✓ | ✓ |  | ✓ | ✓ |  | ✓ |  |  |  | ✓ | ✓ |  | ✓ |
| Payne Riches et al [36], 2021 | ✓ |  |  |  | ✓ | ✓ | ✓ |  | ✓ |  |  |  |  | ✓ | ✓ |  |  |  |  | ✓ | ✓ |
| Sun et al [24], 2020 |  | ✓ |  | ✓ |  | ✓ | ✓ | ✓ | ✓ | ✓ | ✓ |  | ✓ |  |  | ✓ | ✓ | ✓ |  |  |  |
| Zha et al [32], 2020 |  |  | ✓ | ✓ |  |  | ✓ | ✓ | ✓ |  |  |  |  |  |  | ✓ |  |  |  |  |  |
| Zhang et al [22], 2022 |  |  |  |  |  |  |  | ✓ | ✓ |  |  |  |  |  |  | ✓ |  |  |  |  | ✓ |
| Zhang et al [23], 2023 | ✓ |  | ✓ | ✓ |  |  | ✓ | ✓ | ✓ | ✓ | ✓ |  | ✓ | ✓ |  | ✓ |  | ✓ |  |  | ✓ |

^a^✓: Reported in intervention.

**
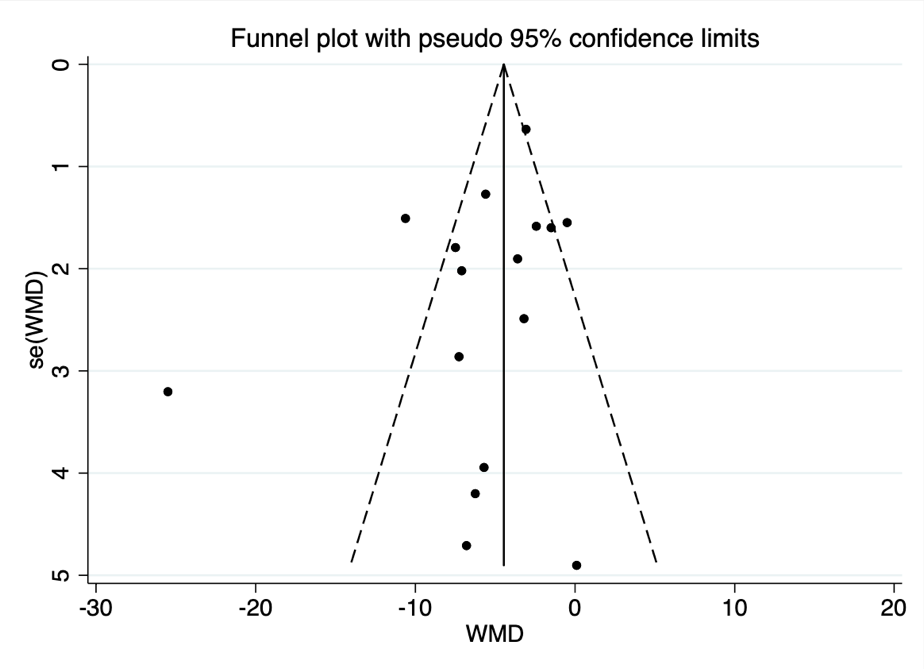
Figure S1. Funnel plot for risk of publication bias.**

**Figure S2. Subgroup analysis of factors mHealth app intervention addressed.**


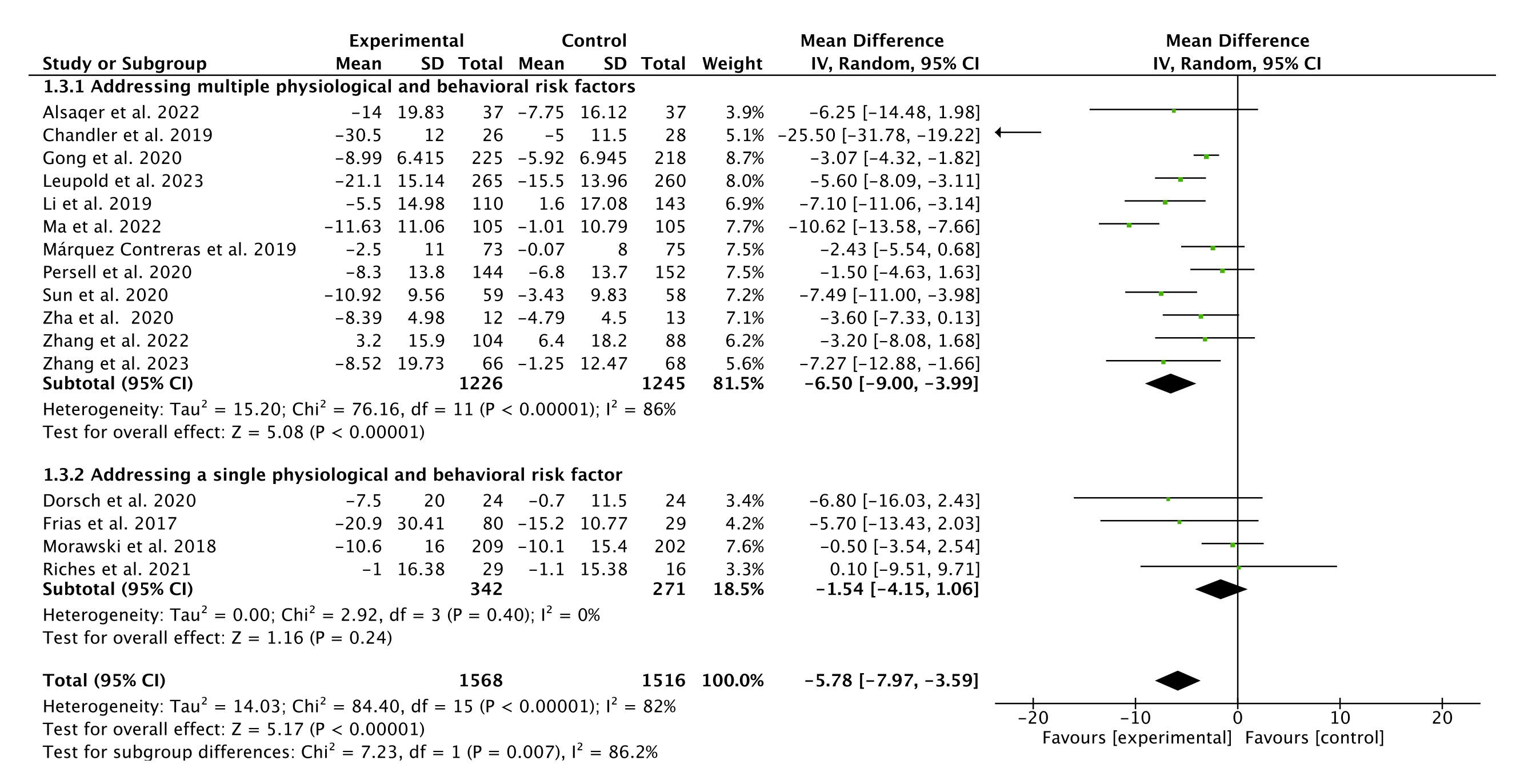


**Figure S3. Subgroup analysis of theoretical foundation of mHealth app intervention.**


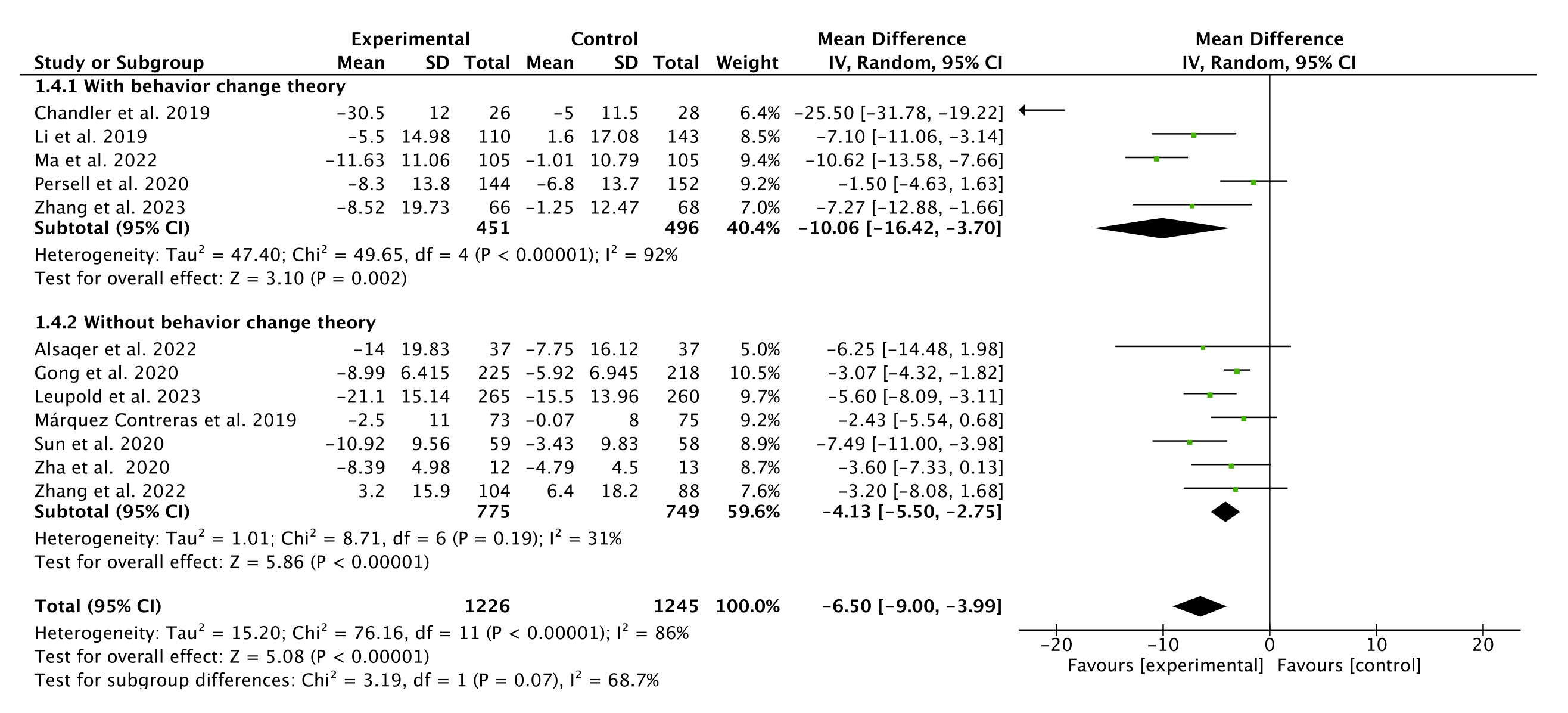


**Figure S4. Subgroup analysis of duration of mHealth app intervention.**


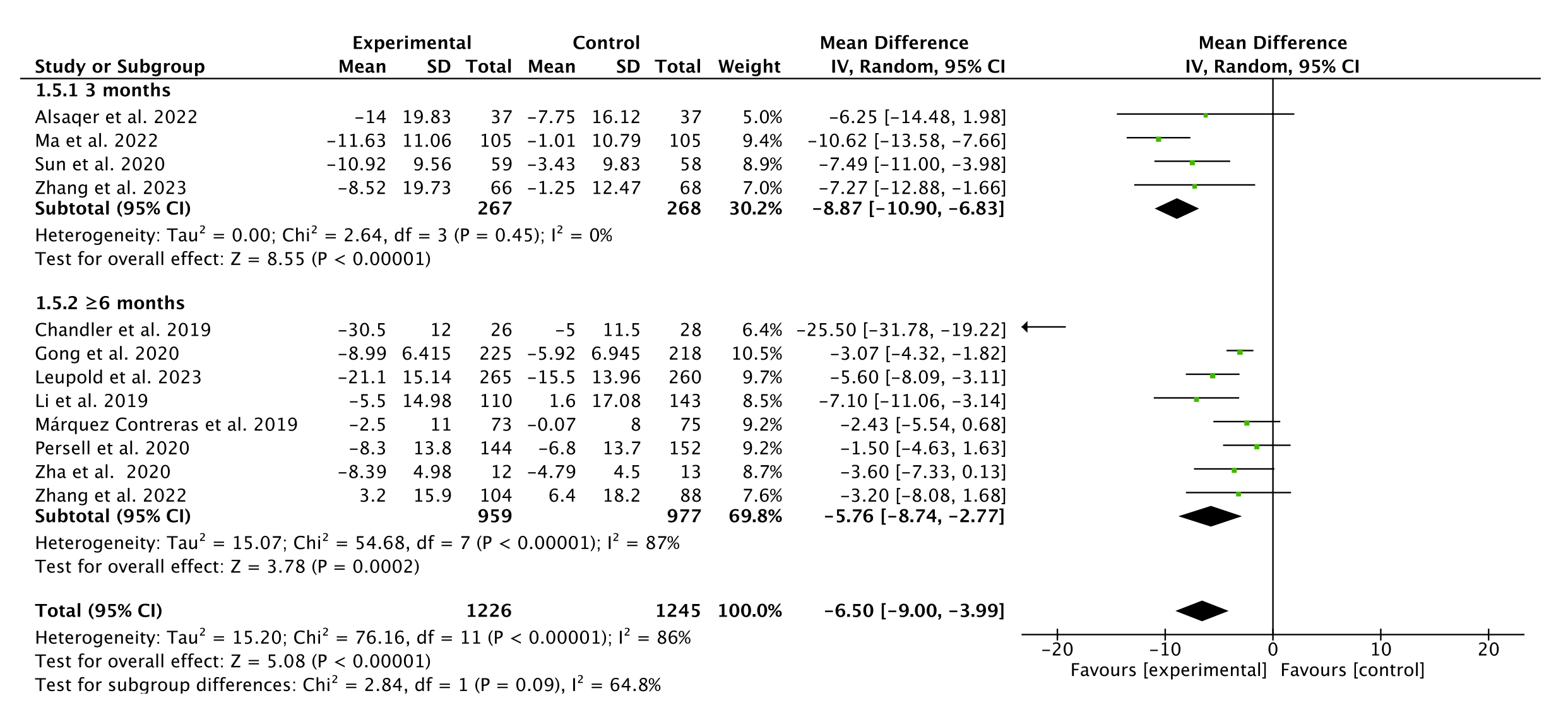


**Figure S5. Subgroup analysis of the number of BCTs used in mHealth app intervention.**


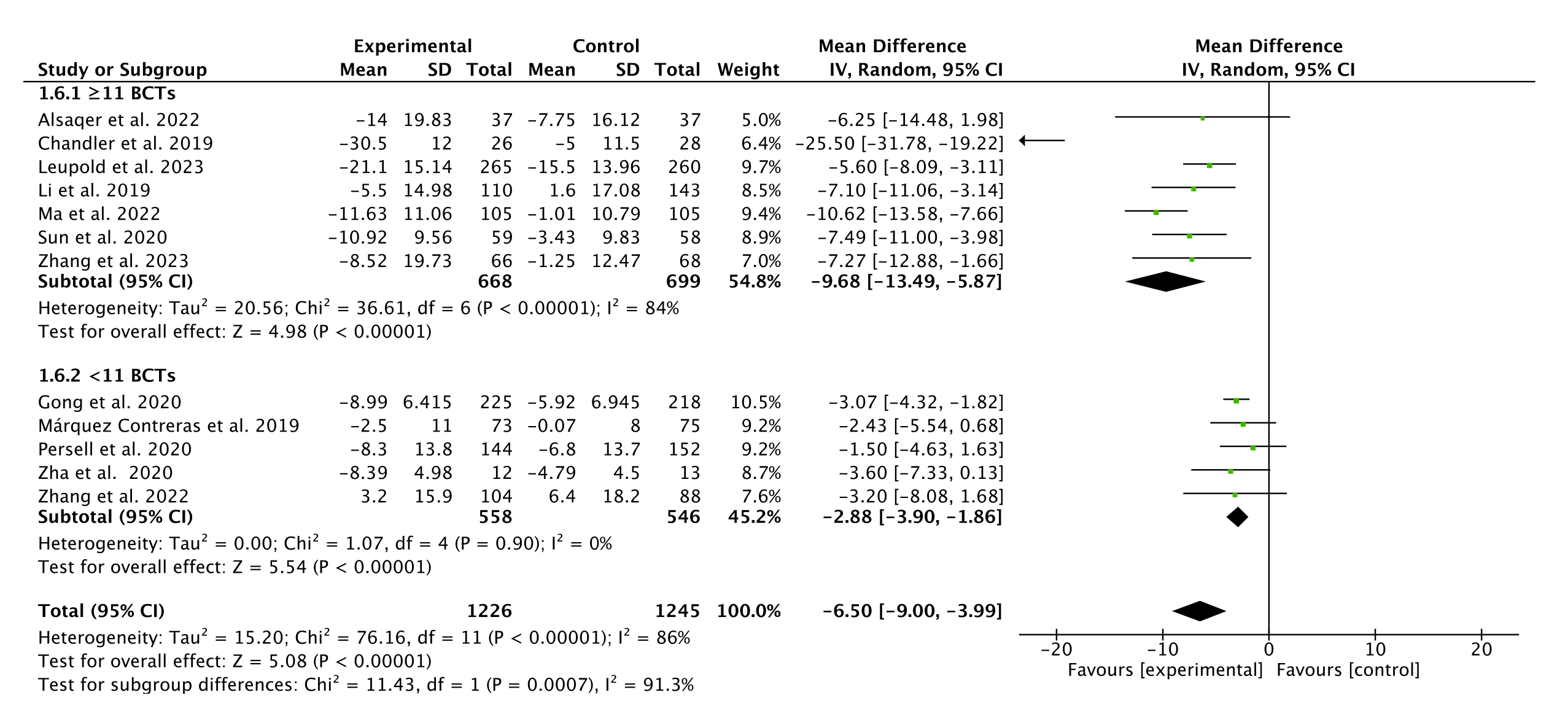


**Reference**

22. Zhang Y, Tao Y, Zhong Y, Thompson J, Rahmani J, Bhagavathula AS, Xu X, Luo J. Feedback based on health advice via tracing bracelet and smartphone in the management of blood pressure among hypertensive patients: a community-based RCT trial in Chongqing, China. Medicine (Baltimore) 2022 Jul 15; 101(28):e29346

23. Zhang YT, Tan XD, Wang Q. Effectiveness of a mHealth intervention on hypertension control in a low-resource rural setting: a randomized clinical trial. Front Public Health 2023 Mar 1; 11:1049396

24. Sun YQ, Jia YP, Lv JY, Ma GJ. The clinical effects of a new management mode for hypertensive patients: a randomized controlled trial. Cardiovasc Diagn Ther 2020 Dec; 10(6):1805-15

25. Li X, Li T, Chen J, Xie Y, An X, Lv Y, Lin A. A WeChat-based self-management intervention for community middle-aged and elderly adults with hypertension in Guangzhou, China: a cluster-randomized controlled trial. Int J Environ Res Public Health 2019 Oct 23; 16(21):4058

26. Ma Y, Cheng HY, Sit JW, Chien WT. The effects of a smartphone-enhanced nurse-facilitated self-care intervention for Chinese hypertensive patients: a randomised controlled trial. Int J Nurs Stud 2022 Oct; 134:104313

27. Gong K, Yan YL, Li Y, Du J, Wang J, Han Y, Zou Y, Zou XY, Huang H, She Q. Mobile health applications for the management of primary hypertension: a multicenter, randomized, controlled trial. Medicine (Baltimore) 2020 Apr; 99(16):e19715

28. Chandler J, Sox L, Kellam K, Feder L, Nemeth L, Treiber F. Impact of a culturally tailored mHealth medication regimen self-management program upon blood pressure among hypertensive Hispanic adults. Int J Environ Res Public Health 2019 Apr 06; 16(7):1226

29. Frias J, Virdi N, Raja P, Kim Y, Savage G, Osterberg L. Effectiveness of digital medicines to improve clinical outcomes in patients with uncontrolled hypertension and type 2 diabetes: prospective, open-label, cluster-randomized pilot clinical trial. J Med Internet Res 2017 Jul 11; 19(7):e246

30. Morawski K, Ghazinouri R, Krumme A, Lauffenburger JC, Lu Z, Durfee E, Oley L, Lee J, Mohta N, Haff N, Juusola JL, Choudhry NK. Association of a smartphone application with medication adherence and blood pressure control: the MedISAFE-BP randomized clinical trial. JAMA Intern Med 2018 Jun 01; 178(6):802-9

31. Persell SD, Peprah YA, Lipiszko D, Lee JY, Li JJ, Ciolino JD, Karmali KN, Sato H. Effect of home blood pressure monitoring via a smartphone hypertension coaching application or tracking application on adults with uncontrolled hypertension: a randomized clinical trial. JAMA Netw Open 2020 Mar 02; 3(3):e200255

32. Zha P, Qureshi R, Porter S, Chao YY, Pacquiao D, Chase S, O'Brien-Richardson P. Utilizing a mobile health intervention to manage hypertension in an underserved community. West J Nurs Res 2020 Mar; 42(3):201-9

33. Dorsch MP, Cornellier ML, Poggi AD, Bilgen F, Chen P, Wu C, An LC, Hummel SL. Effects of a novel contextual just-in-time mobile app intervention (LowSalt4Life) on sodium intake in adults with hypertension: pilot randomized controlled trial. JMIR Mhealth Uhealth 2020 Aug 10; 8(8):e16696

34. Bozorgi A, Hosseini H, Eftekhar H, Majdzadeh R, Yoonessi A, Ramezankhani A, Mansouri M, Ashoorkhani M. The effect of the mobile “blood pressure management application” on hypertension self-management enhancement: a randomized controlled trial. Trials 2021 Jun 24; 22:413

35. Najafi Ghezeljeh T, Sharifian S, Nasr Isfahani M, Haghani H. Comparing the effects of education using telephone follow-up and smartphone-based social networking follow-up on self-management behaviors among patients with hypertension. Contemp Nurse 2018 Mar 05; 54(4-5):362-73

36. Payne Riches S, Piernas C, Aveyard P, Sheppard JP, Rayner M, Albury C, Jebb SA. A mobile health salt reduction intervention for people with hypertension: results of a feasibility randomized controlled trial. JMIR Mhealth Uhealth 2021 Oct 21; 9(10):e26233

37. Márquez Contreras E, Márquez Rivero S, Rodríguez García E, López-García-Ramos L, Carlos Pastoriza Vilas J, Baldonedo Suárez A, Gracia Diez C, Gil Guillén V, Martell Claros N. Specific hypertension smartphone application to improve medication adherence in hypertension: a cluster-randomized trial. Curr Med Res Opin 2018 Dec 05; 35(1):167-73

38. Leupold F, Karimzadeh A, Breitkreuz T, Draht F, Klidis K, Grobe T, Weltermann B. Digital redesign of hypertension management with practice and patient apps for blood pressure control (PIA study): a cluster-randomised controlled trial in general practices. eClinicalMedicine 2023 Jan; 55:101712

39. Kario K, Nomura A, Kato A, Harada N, Tanigawa T, So R, Suzuki S, Hida E, Satake K. Digital therapeutics for essential hypertension using a smartphone application: a randomized, open‐label, multicenter pilot study. J Clin Hypertens 2021 Jan 23; 23(5):923-34

40. Abu-El-Noor N, Aljeesh Y, Bottcher B, Abu-El-Noor M. Impact of a mobile phone app on adherence to treatment regimens among hypertensive patients: a randomised clinical trial study. European Journal of Cardiovascular Nursing. Eur J Cardiovasc Nurs 2021 Jun; 20(5):428-35

41. Alsaqer K, Bebis H. Self-care of hypertension of older adults during COVID-19 lockdown period: a randomized controlled trial. Clin Hypertens 2022 Jul 15; 28:21
